# Influence of design of dentist’s chairs on body posture for dentists with different working experience

**DOI:** 10.1186/s12891-021-04334-1

**Published:** 2021-05-19

**Authors:** F. Huppert, W. Betz, C. Maurer-Grubinger, F. Holzgreve, L. Fraeulin, N. Filmann, D. A. Groneberg, D. Ohlendorf

**Affiliations:** 1 Institute of Occupational Medicine, Social Medicine and Environmental Medicine, Goethe-University Frankfurt/Main, Theodor-Stern-Kai 7, Building 9A, 60590 Frankfurt/Main, Germany; 2 Institute of Dentistry, Goethe-University, Frankfurt/Main, Theodor-Stern-Kai 7, 60590 Frankfurt am Main, Germany; 3 Institute of Biostatistics and Mathematical Modeling, Goethe-University, Frankfurt/Main, Theodor-Stern-Kai 7, Building 11, 60590 Frankfurt/Main, Germany

**Keywords:** Posture analysis, Dentists, Musculoskeletal problems, dentist’s chair design

## Abstract

**Background:**

Musculoskeletal disorders (MSD) are a common health problem among dentists. Dental treatment is mainly performed in a sitting position. The aim of the study was to quantify the effect of different ergonomic chairs on the sitting position. In addition, it was tested if the sitting position of experienced workers is different from a non-dental group.

**Methods:**

A total of 59 (28 m/31f) subjects, divided into two dentist groups according to their work experience (students and dentists (9 m/11f) < 10 years, dentists (9 m/10f) ≥ 10 years) and a control group (10 m/10f) were measured. A three-dimensional back scanner captured the bare back of all subjects sitting on six dentist’s chairs of different design. Initially, inter-group comparisons per chair, firstly in the habitual and secondly in the working postures, were carried out. Furthermore, inter-chair comparison was conducted for the habitual as well as for the working postures of all subjects and for each group. Finally, a comparison between the habitual sitting posture and the working posture for each respective chair (intra-chair comparison) was conducted (for all subjects and for each group). In addition, a subjective assessment of each chair was made.

For the statistical analysis, non-parametric tests were conducted and the level of significance was set at 5%.

**Results:**

When comparing the three subject groups, all chairs caused a more pronounced spinal kyphosis in experienced dentists. In both conditions (habitual and working postures), a symmetrical sitting position was assumed on each chair.

The inter-chair comparisons showed no differences regarding the ergonomic design of the chairs. The significances found in the inter-chair comparisons were all within the measurementerror and could, therefore, be classified as clinically irrelevant.

The intra-chair comparison (habitual sitting position vs. working sitting position) illustrated position-related changes in the sagittal, but not in the transverse, plane. These changes were only position-related (forward leaned working posture) and were not influenced by the ergonomic sitting design of the respective chair. There are no differences between the groups in the subjective assessment of each chair.

**Conclusions:**

Regardless of the group or the dental experience, the ergonomic design of the dentist’s chair had only a marginal influence on the upper body posture in both the habitual and working sitting postures. Consequently, the focus of the dentist’s chair, in order to minimize MSD, should concentrate on adopting a symmetrical sitting posture rather than on its ergonomic design.

**Supplementary Information:**

The online version contains supplementary material available at 10.1186/s12891-021-04334-1.

## Background

It has been shown that dentists who work in a sitting position are more likely to report severe lower back pain than those who alternate between sitting and standing [1, 2]. Since the introduction of treatment on the reclined patient in dentistry in the 1960s, dentistry has changed from being usually performed in a standing position to being executed mostly in a sitting position [3, 4]. An increased working time in a sitting position may be related to an increasing number of musculoskeletal complaints (MSD), which represent a major impairment [5–17]. Numerous studies [5–15, 17] have shown that between 64 and 93% of subjects surveyed (dentists, dental students, dental hygienists and dental assistants) stated that they suffer from MSD. The most affected areas are the neck (19.8–85%), the shoulders (20–65%) and, in particular, the back area (36.3–60.1%) [12]. Lietz et al. [18] confirmed these results in their review. Headaches, numbness, paraesthesia and complaints of the hands or knees are also often described as side effects [1, 11, 19]. Only irregular correlations between the reported complaints with regard to age differences and the work experience of dentists can be found [8, 20–22]. In general, these results are globally reported [5–15].

Further factors such as an unfavorable posture [5, 12, 21, 23, 24], psychological stress [1, 11] or a lack of physical activity [25] can lead to a heavy physically demanding body strain. A highly physically demanding working posture is often be assumed while gaining insight into the patient’s mouth; this means that the dentist sits on the right-hand side of the patient with a left-sided rotation, right-sided lateral flexion and ventral inclination of the upper body [15, 26, 27]. Furthermore, the hip and knee flexion include angle values of less than 90°, while the head is bent ventrally and rotated to the left. The right arm is raised up to 90°, rotated inwards with the forearm strongly flexed, while the left arm is abducted 60° and rotated inwards [28]. The described posture has been observed in right-handed dentists, but also applies to left-handed dentists in the mirror-inverted positions. Consequently, it can be assumed that this constantly same working posture over many days, weeks, months and even years leads to the development of biomechanical strategies in order to fulfill the work requirements.

This can lead to muscular fatigue and strain on the peripheral nervous system due to recurring movements and static positions held for long periods of time. Over time, these can also lead to pathological changes in the musculoskeletal system and the spine [1, 19, 21, 29, 30]. Many factors can influence the working posture including the equipment of the workplace [31], the selection of working instruments, the type of working technique, patient positioning, the lighting conditions prevailing at work or even the dentist’s chair [32–35].

As a result of these work-related complaints the field of dental ergonomics has generally received increasing attention in recent years. In the field of dental ergonomics, efforts are being made to reduce these prevailing physical strains by, among other things, attempting to modify the dentist’s chair layout. According to the manufacturer, the dentist’s chair should allow the user to sit in a stable, relaxed position with a straight back, without twisting the spine or turning the head and with a good view of the working area [27, 36]. Ergonomically shaped dentist’s chairs differ mainly in the design of their seat and backrest.

Related studies such as those by Gandavadi et al. [33] and Dable et al. [34] found an acceptable working posture for the saddle seat (final risk scores in the range of 2–3) compared to a conventional comparison chair (final risk scores in the range of 3–7) by using the Rapid Upper Limb Assessment (RULA) to evaluate different dentist’s chairs.

Haddad et al. [37] and Fiedler [38] used EMG to determine the muscle activity during sitting. Fiedler [38] examined the activity of various muscles (M. erector spinae, *M. deltoideus*, M. biceps brachii, M. triceps brachii, M. sternocleidomastoideus, M. trapezius (pars descendens and pars horizontalis) and *M. rectus* abdominis) while sitting on six different chairs in five different posture positions. Although Haddad [37] was able to demonstrate chair-specific muscle activity (M. trapezius pars descendens an pars horizontalis) between two different dentist’s chairs while performing examination tasks of the lower jaw, Fiedler [38] refuted these results since the muscle activity was almost identical on each chair despite the different layouts.

Parameters such as the pelvic rotation on different dentist’s chairs were analyzed by De Bruyne et al. [32] using strain gauges. Different sagittal pelvic tilts were observed with simultaneous changes in lumbar pressure and trunk muscle activity depending on the chair design used.

Apart from the numerous examination methods mentioned above, there have been no analyses to date which investigate the influence of the ergonomic design of dentist’s chairs on the upper body posture and the resulting changes in the spinal column. Since not every method can depict all changes, with the help of different examiniation methods it is possible to obtain comprehensive insights into the changes from different perspectives. However, a suitable method to investigate such potential influences is videorasterstereography [39–44]. This method has been successfully used to examine the upper body posture of musicians sitting on musician chairs of a different ergonomic layout [45]. In order to correlate results obtained with different methods in the future, further studies are necessary.

Thus, six exemplary dentist’s chairs were examined in this study. The aim was to clarify whether dentists adopted a more asymmetrical sitting posture on the chairs, compared to a control group, due to their profession and whether the professional experience within the dentists made a visible difference in their posture. Regardless of the choice of occupation, it was examined whether the various chair designs showed clinically relevant differences, firstly, in the habitual sitting posture and, secondly, in a simulated working posture. Furthermore, the two sitting postures were compared.

The hypotheses to be tested are:

Hypothesis 1: Experienced and less experienced dentists show asymmetries in the shoulder and pelvic region when habitual sitting on different dentist’s chairs compared to the control group.

Hypothesis 2: Due to the biomechanical strategies acquired during working life, the spine and shoulder position worsened (less symmetrical) with increasing dental work experience when sitting on the examined dentist’s chairs in working posture.

Hypothesis 3: When habitually sitting on the saddle chair, the straightening of the pelvis results in an increased kyphosis and lordosis angles compared to the other chairs.

Hypothesis 4: The comparison of the habitual with the working sitting posture results in postural differences in the cervical and thoracic regions.

## Methods

### Subjects

Fifty nine subjects (31w/28 m) aged 24 to 69 years (37.8 years ±15.1 years) with an average height of 174.7 cm (± 7.94 cm) and an average body weight of 71.03 kg (± 12.2 kg) were divided into three groups. Accordingly, group 1, the control group, consisted of healthy subjects (via self-assessment) without any dental work experience, group 2 consisted of dentists or dentistry students with work experience of less than 10 years and group 3 comprised dentists with work experience of more than 10 years (Table 1). While there is a significant difference between groups 1 and 3 as well as 2 and 3 with regard to age, no differences could be demonstrated with regard to height and weight. The difference in age between groups 1 and 2 and group 3 confirms the statement that age is related to work experience.

**Table 1:** Descriptive data (group distribution, gender, age, height, body weight) of all test persons. Significant differences between two groups are marked with the same superscript letter (a or b)

	Group 1	Group 2	Group 3
Group description	No dental education	Students of dentistry / dentists < 10 years professional experience	Dentists ≥10 years professional experience
Gender	20 (10w/10 m)	20 (11w/9 m)	19 (10w/9 m)
Age (years)	23–52 ^a^ (29.90 ± 8.04)	24–32 ^b^ (26.40 ± 2.06)	45–69 ^a,b^ (56.42 ± 6.88)
Body height (cm)	160–193 173.40 ± 8.01	176.65 ± 8.31	173.63 ± 7.31
Weight (kg)	50–100 71.08 ± 12.79	68.65 kg ± 13.16	73.53 ± 10.07

The following criteria applied as exclusion criteria for the study: undergoing surgery within the last 6 months, scoliosis, torn ligaments, acute herniated discs, spinal surgery, trauma lesions, genetic neurological or muscular diseases, influence of medication from analgesics or muscle relaxants and other conditions affecting the spine or musculature.

All test persons had given their written consent to participate in this study. The study was approved by the ethics committee of the Medical Faculty of the Goethe-University (Nr. 140/10). All methods were performed in accordance with the relevant guidelines and regulations.

### Measurement system

#### Three-dimensional back scan

The upper body position was measured by using the ABW BodyMapper (ABW GmbH, Frickenhausen, Germany), a light optical device based on videorasterstereography. The maximum frame rate of this device is 50 frames/sec with a depth resolution of 1/100 mm, while the error of measurement, according to the manufacturer, should be < 1 mm and for repeated measurements less than 0.5 mm. Six light-reflecting, self-adhesive skin markers (of 1 cm diameter) were attached to six anatomical landmarks (vertebra prominens, lower scapula angle right and left, posterior superior iliac spine left and right and the sacrum point (rima ani) (Fig. 2) [46, 47] directly on the back of the test person prior to the measurements.

Videorasterstereography has already been confirmed as a suitable method for recording upper body posture [39–44] and, therefore, was used in further studies to obtain representative standard values for young women (21–30 years) [48], young men (18–35 years) [49] and middle-aged men (41–50 years) [50]. A detailed description of the spine parameters examined can be found in [51].

### Dentist’s chairs

**Fig. 1:**
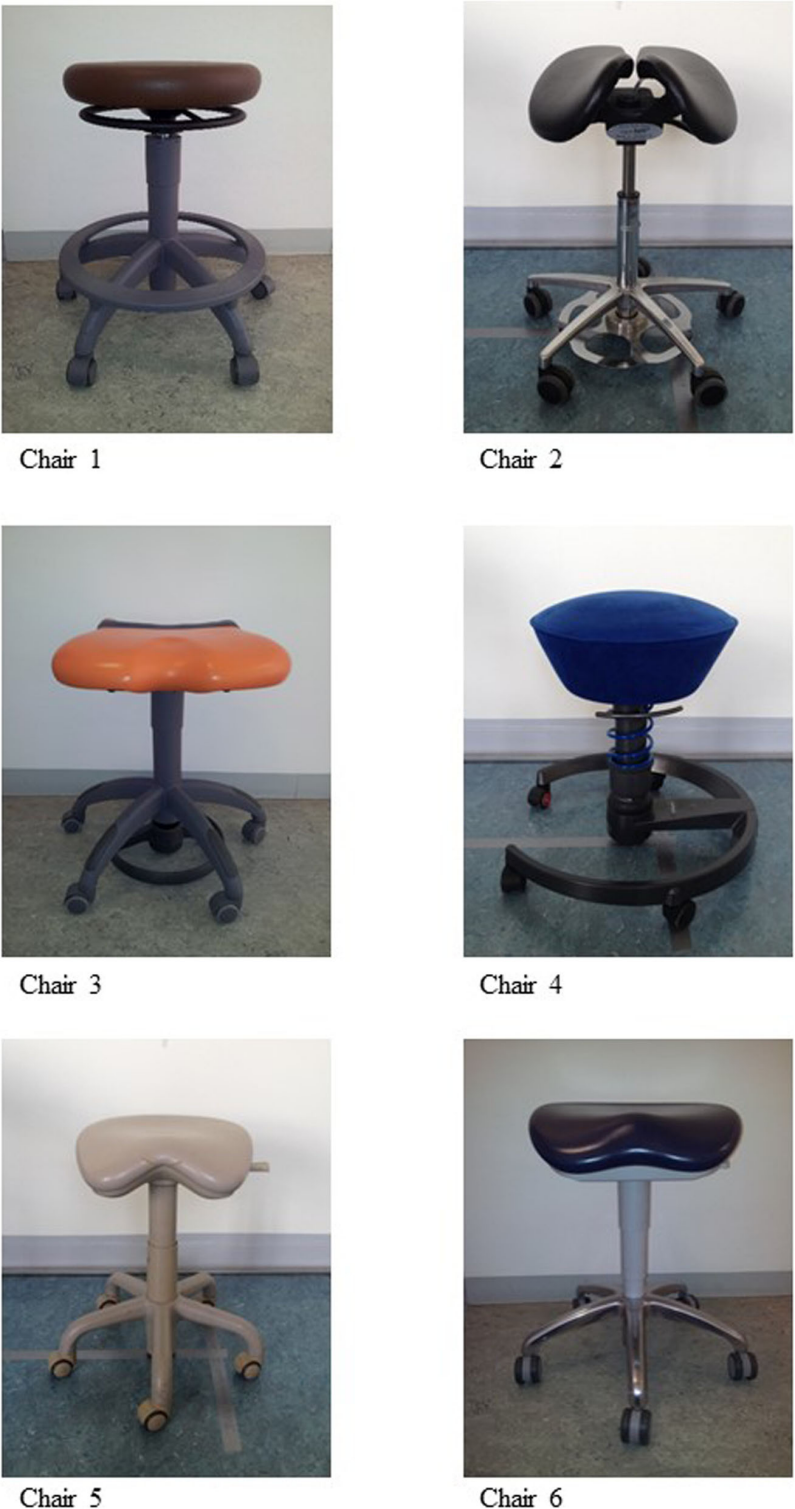
Types of chairs used: 6 different ergonomic chairs used by professional dentists were measured. Different dental working chairs

Six different chairs were selected for this study. They differed in their design and underlying ergonomics; these are summarized in Table 2 and all sitting concepts are additionally illustrated in Fig. 1.

**Table 2:** Characteristics of the six chairs used: special attributes, sitting area and height of sitting position

Chair	Abbreviation	Special attributes/ backrest	Sitting area	Height of sitting position (knee ankle)	Company, chair name
Chair 1	Sib	no backrest	disc-shaped, horizontal	height adjustable	Sirona, CARL
Chair 2	Sal	no backrest	saddle-shaped, two parts mutually adjustable, adjustable inclination	height adjustable manufacturer information: 135° knee angle	Salli, Saddle chair
Chair 3	Sio	backrest removed	ventrally inclined, flexible in the front part	height adjustable	Sirona, Hugo Freehand
Chair 4	Sw	no backrest	hemispherical, resilient, flexible	height adjustable weight adjustable, spring tension adjustable, lateral deflection adjustable	Aeris, Swopper
Chair 5	Kg	backrest removed	ventrally inclined, central elevation	height adjustable	KaVo, Physioform 5005
Chair 6	Kb	backrest removed	ventral inclination to max. 15°, central elevation	height adjustable	KaVo, Evo

Chair 1,2,3, 5 and 6 were chosen since they are the models commonly used in German dental practices and universities. Only chair 4, the Swopper, is not designed for the field of dentistry. It is an ergonomically designed chair whose construction is flexible enough to allow “moving sitting” (bouncing). Body muscles can thus be moved unconsciously, actively reducing a static sitting posture.

### Measurement protocol

The investigation was conducted at the Institute of Occupational Medicine, Social Medicine and Environmental Medicine at Goethe-University Frankfurt/Main (Germany). One measurement took about 2 s. Since we performed a high number of measurements on the 6 chairs, the measurement of each subject took approx. 1 hour.

Based on the questionnaire used in the study on musicians’ chairs [45], the subjects in this study were asked about their subjective evaluation of the individual chairs by using school grades. When grading the chairs, each mark from 1 to 6 does not necessarily have to be awarded once, but several chairs could also receive the same marks.

Prior to the initial study, the questionnaire was evaluated in 10 dental students.

In this context, the following questions, among others, should be answered in relation to each chair: How did you like the chair designs you just tried? What did you particularly like about the best chair? Here, the three main criteria mentioned by the test persons were the comfort, the clearly defined sitting position of some chairs and the ergonomic design.

Prior to the body posture measurements being taken, the backrests of chairs 3, 5 and 6 (Sirona Hugo freehand, KaVo Physioform 5005 and KaVo Evo) were removed for the duration of the measurements in order to enable the measurement of the back down to the landmark rima ani. Dable et al. [34] confirmed that the backrest has no influence on the sitting behavior.

**Fig. 2:**
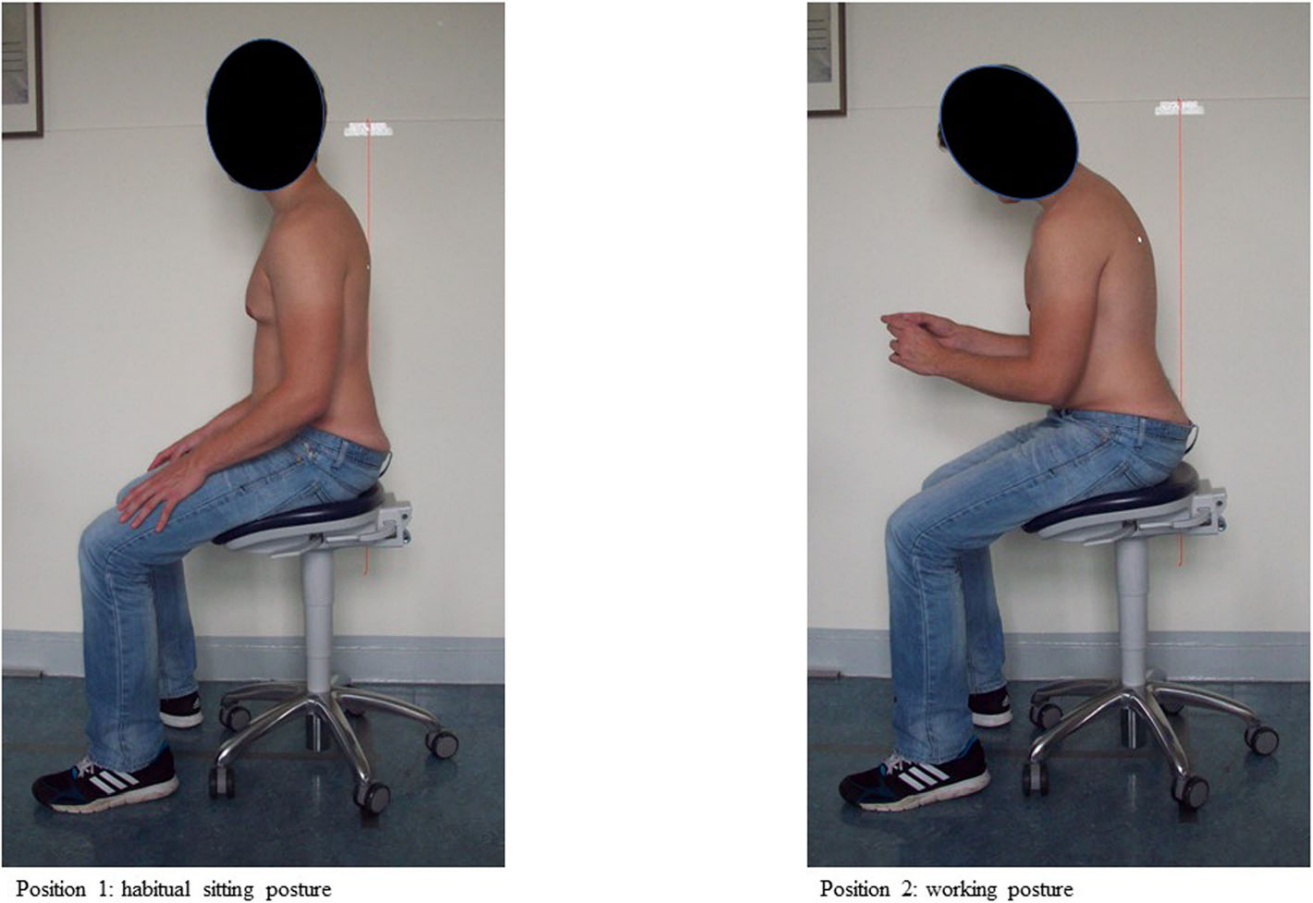
Both measurement conditions. The left side shows the habitual sitting posture (condition 1), while the right side shows the working posture (condition 2). Six attached anatomical landmarks, habitual sitting posture (position 1) and simulated dental working posture (position 2)

The order of the chairs and the sitting postures were randomized. Each subject was measured in two different sitting postures (the habitual sitting posture and the simulated dental working posture) on all six chairs (Fig. 2). For the habitual sitting posture, the test person sat upright while the legs were placed vertically with both feet on the floor and the hands placed on the thighs. For the dental working posture, the subject was asked to adopt the following posture: the upper body was tilted forwards by approximately 10° in the hip joint. The test person held an object with both hands and tilted their head downwards to be able to look at it. The distance between the eyes and the object was approximately 30–40 cm. The seat height was adjusted so that an angle of between 90 and 135° was assumed in the knee joint, according to the manufacturer’s recommendation. All angles to be adopted were checked with the help of a goniometer [3, 27, 52, 53].

Three consecutive measurements were performed from which the mean value was calculated.

Additionally a subjective assessment of chair comfort was conducted which based on the school grading system, with 1 representing the best and 6 the worst rating.

### Statistical data analysis

The program “BiAS 11.08” (Epsilon-Verlag, Darmstadt, Germany) was used for the statistical analysis. Firstly, normality assumptions were tested by the Kolmogoroff-Smirnoff-Lilliefors test. Since the data did not show a normal distribution, non-parametric tests were used.

Secondly, in order to compare all chairs for each back parameter, either in the habitual sitting position or in the dental working position, the Friedman-test was carried out with subsequent multiple pair comparisons.

All three groups were compared with the Kruskal-Wallis test followed by multiple pair comparisons. To compare both postures, the Wilcoxon-matched-pairs-test was used.

All *p*-values were corrected by Bonferroni-Holm correction. The subjective assassement of chair comfort was calculated by the Chi^2^ test for the contingency table.

The significance level was 5%.

## Results

Table 3 shows the median, minimum and maximum for all the parameters of the upper body posture (spinal column, shoulder and pelvic parameters) during the habitual sitting of either all subjects or of each group individually. Table 4 contains equivalent values for the working posture.

**Table 3:** Habitual sitting posture. Presentation of median, minimum and maximum of all measurement parameters for all test persons and for each individual group. Chair 1: Sirona CARL (Sib), Chair 2: Salli Saddle chair (Sal), Chair 3: Sirona Hugo Freehand (Sio), Chair 4: Aeris Swopper (Sw), Chair 5: KaVo Physioform 5005 (Kg), Chair 6: KaVo Evo (Kb)

Habitual sitting posture		Chair 1 Sib		Chair 2 Sal		Chair 3 Sio		Chair 4 Sw		Chair 5 Kg		Chair 6 Kb	
	Groups	Median	Minimum/Maximum	Median	Minimum/Maximum	Median	Minimum/Maximum	Median	Minimum/Maximum	Median	Minimum/Maximum	Median	Minimum/Maximum
**Spine parameter**													
Trunk length D (mm) SRDL	All groups	490.72	470.84/ 510.22	484.29	465.99/ 509.74	488.92	469.79/ 511.52	488.71	467.84/ 509.79	491.82	471.39/ 510.73	491.72	475.72/ 517.26
	1	504.60	471.93/ 529.19	499.34	471.90/ 523.45	498.18	472.66/ 527.29	498.62	471.68/ 527.41	498.53	475.03/ 532.33	500.80	474.52/ 529.96
	2	493.78	475.17/ 518.14	488.07	468.40/ 510.06	492.53	473.10/ 519.12	493.04	471.69/ 518.71	494.08	474.71/ 516.30	494.23	477.53/ 521.79
	3	480.40	461.49/ 492.80	472.09	460.27/ 498.38	482.68	460.86/ 494.56	475.68	457.08/ 492.40	478.35	464.78/ 498.93	487.64	462.86/ 505.96
Trunk length S (mm) SRLS	All groups	534.73	516.75/ 556.13	528.27	509.47/ 548.39	534.25	515.62/ 551.89	532.97	512.44/ 554.46	535.59	514.34/558.62	540.06	516.90/ 560.19
	1	536.02	523.54/ 563.12	534.89	516.89/ 557.37	535.25	527.41/ 553.50	534.19	521.55/ 558.79	538.70	519.85/ 565.54	543.18	524.98/ 569.79
	2	548.38	527.26/ 562.39	537.95	525.09/ 550.39	540.41	523.93/ 562.94	546.89	522.63/ 562.53	542.03	526.32/ 566.14	549.69	531.23/ 568.25
	3	517.25	505.25/ 536.96	513.81	501.03/ 536.55	515.62	503.23/ 538.00	514.24	501.88/ 536.03	521.89	503.8/ 538.63	520.69	509.13/ 545.77
Sagittal trunk decline (°) SSRN	All groups	−7.03	−8.71/ −5.32	−5.78	−7.33/ −4.09	−7.46	−8.93/ −6.12	−6.62	−8.66/ −4.47	−7.26	−9.31/ −5.41	− 7.57	− 8.80/ − 5.25
	1	−6.56	− 7.67/ −3.81	− 5.82	− 7.27/ − 4.23	− 7.20	− 8.78/ − 6.20	− 6.44	− 7.83/ −3.59	− 6.55	− 8.07/ − 4.36	− 7.20	−8.42/ − 4.80
	2	− 7.05	− 9.92/ − 5.37	− 5.78	−7.33/ − 3.71	− 7.66	−9.25/ − 6.26	− 7.48	− 8.98/ − 4.16	− 7.70	−10.41/ − 5.55	− 7.78	−9.80/ − 4.47
	3	− 7.16	−9.33/ − 5.32	− 5.52	−7.44/ − 4.43	− 7.59	−9.36/ − 5.86	− 6.62	− 8.45/ − 5.38	− 8.72	− 9.88/ − 5.95	− 7.77	−9.34/ − 6.00
Frontal trunk decline (°) SFRN	All groups	0.73	0.15/ 1.36	0.54	−0.38/ 1.59	0.48	− 0.07/ 1.46	0.96	0.24/ 1.50	0.78	0.14/ 1.70	0.73	−0.04/ 1.38
	1	1.26	0.41/ 2.05	0.79	−0.14/ 1.68	0.59	−0.07/ 1.92	1.04	0.26/ 1.75	0.89	−0.08/ 2.07	1.14	0.16/ 1.51
	2	0.70	−0.06/ 1.41	0.60	−0.37/ 1.59	0.72	0.11/ 1.61	1.11	0.41/ 1.57	0.64	0.23/ 1.64	0.66	0.12/ 1.45
	3	0.30	−0.60/ 0.76	0.33	−0.43/ 1.01	0.25	− 0.33/ 1.33	0.72	−0.36/ 1.00	0.80	− 0.50/ 1.49	0.52	−0.69/ 1.31
Axis decline (°) SAA	All groups	−0.10	− 1.70/ 1.15	− 0.23	−1.98/ 0.95	− 0.45	−1.64/ 0.64	− 0.73	−1.81/ 0.87	− 0.15	−1.38/ 1.10	0.02	− 1.37/ 1.34
	1	0.75	−1.09/ 2.14	0.36	− 1.86/ 0.99	0.02	−1.40/ 0.77	− 0.02	−1.31/ 0.89	0.20	−0.96/ 1.24	0.73	− 1.02/ 1.63
	2	− 0.07	− 1.68/ 0.85	− 0.96	− 2.14/ 1.04	− 0.41	− 1.00/ 0.46	− 0.78	−1.80/ 0.85	− 0.18	−1.91/ 0.98	− 0.29	−1.55/ 1.31
	3	−0.64	− 2.25/ 0.24	− 0.83	−1.98/ 0.47	− 1.01	−2.14/ 0.23	− 1.18	−2.27/ 0.78	− 0.82	− 1.96/ 0.84	− 0.25	−1.58/ 0.70
Thoracic bending angle (°) STBW	All groups	16.56	12.84/ 19.08	16.83	13.64/ 19.20	18.13	14.64/ 20.23	16.61	13.66/ 19.33	16.16	13.37/ 19.89	17.07	13.23/ 19.52
	1	15.81	12.55/ 18.27	16.81	13.72/ 18.83	15.60	13.98/ 19.57	15.79	14.09/ 16.87	15.24	12.83/ 17.73	16.42	12.84/ 18.06
	2	13.88	12.55/ 18.81	15.70	13.16/ 17.88	17.47	13.20/ 19.97	16.21	13.02/ 17.44	15.67	12.73/ 19.16	14.80	12.53/ 19.22
	3	18.87	14.92/ 22.26	19.10	13.37/ 22.05	19.56	14.69/ 23.14	18.32	13.47/ 24.01	19.14	15.71/ 23.26	19.52	14.46/ 23.17
Lumbar bending angle (°) SLBW	All groups	7.81	5.67/ 10.44	8.71	6.29/ 10.75	7.48	4.84/ 10.69	6.87	5.00/ 10.47	7.62	4.93/ 10.70	8.60	6.00/ 10.77
	1	7.93	5.48/ 10.29	7.99	4.55/ 9.10	6.43	4.22/ 8.18	6.11	3.84/ 9.93	7.09	3.57/ 9.36	8.00	4.47/ 11.58
	2	6.93	5.21/ 10.09	8.27	5.38/ 10.35	6.25	4.62/ 9.96	6.16	4.95/ 9.47	6.75	4.44/ 8.87	7.70	5.41/ 9.91
	3	9.34	6.08/ 13.55	10.61	8.93/ 14.46	9.75	6.59/ 12.52	9.90	6.43/ 13.10	9.79	6.91/ 12.94	9.39	8.41/ 10.77
Standard deviation lateral deviation (mm) SSAS	All groups	3.55	2.81/ 4.68	3.84	2.10/ 5.89	3.55	2.40/ 5.45	3.39	2.26/ 4.79	3.31	2.20/ 4.91	3.66	2.34/ 5.52
	1	3.71	2.81/ 5.07	3.21	1.97/ 6.01	3.61	2.34/ 4.86	3.26	2.04/ 5.27	3.20	2.01/ 5.01	3.88	2.34/ 5.16
	2	3.68	2.73/ 4.19	4.42	2.14/ 5.52	3.60	2.76/ 6.07	3.36	2.19/ 3.90	3.23	2.23/ 4.54	3.65	2.02/ 5.49
	3	3.32	2.85/ 5.45	4.43	1.75/ 6.34	3.24	1.94/ 5.49	3.54	2.59/ 4.95	3.43	1.61/ 6.19	3.36	2.57/ 5.64
Maximal lateral deviation (mm) SMSA	All groups	−4.04	−6.20/ 4.64	−3.66	−8.16/ 3.75	− 4.10	− 6.63/ 4.32	−1.80	−5.42/ 6.29	−0.98	− 5.67/ 5.93	−1.76	− 6.22/ 5.58
	1	−2.99	− 6.91/ 6.59	− 3.69	− 6.97/ 4.14	− 0.64	− 4.21/ 6.74	0.19	− 5.04/ 7.96	0.37	− 4.22/ 6.38	0.18	−4.14/ 7.25
	2	− 3.27	−6.06/ 3.06	0.99	−7.52/ 8.34	− 5.19	−6.78/ 0.15	− 0.13	−5.13/ 6.64	2.82	− 5.40/ 6.54	− 1.08	−7.38/ 5.00
	3	−4.63	−6.71/ 5.07	− 6.50	−9.98/ 1.60	− 4.98	−9.91/ 2.15	− 4.67	−7.90/ 1.92	− 4.76	− 11.16/ 1.22	− 4.39	−9.35/ 5.58
Standard deviation rotation (°) SSAR	All groups	3.01	1.79/ 4.66	2.60	2.02/ 4.10	3.07	2.18/ 4.44	3.04	2.13/ 3.82	2.95	2.12/ 4.10	2.83	2.10/ 3.92
	1	3.52	1.79/ 4.90	2.82	2.01/ 4.14	2.71	1.89/ 4.36	2.24	2.02/ 3.28	2.80	1.95/ 4.19	2.88	1.42/ 4.42
	2	2.36	1.74/ 4.62	2.31	1.89/ 4.21	3.22	2.65/ 4.16	3.28	2.05/ 3.80	2.99	2.03/ 3.71	2.47	1.75/ 3.00
	3	3.33	2.49/ 4.47	2.88	2.28/ 3.63	3.10	2.18/ 5.53	3.05	2.28/ 4.65	3.22	2.43/ 5.04	3.23	2.40/ 4.37
Maximal rotation (°) SMR	All groups	4.46	−3.70/ 7.41	3.78	−3.99/ 7.68	5.27	−4.10/ 7.38	5.20	− 3.61/ 7.16	5.03	−1.46/ 6.99	5.05	−2.63/ 6.48
	1	3.88	− 2.61/ 7.78	5.15	− 3.61/ 7.83	4.33	−4.44/ 6.74	4.10	− 3.37/ 5.67	4.63	− 2.50/ 6.19	2.89	−3.78/ 7.13
	2	4.46	−0.83/ 6.97	4.40	− 2.05/ 7.50	6.31	3.70/ 7.66	6.20	3.70/ 7.65	5.89	0.19/ 7.36	5.10	1.54/ 5.97
	3	4.73	−5.42/ 7.13	1.52	−5.35/ 7.93	3.44	−4.11/ 8.94	5.26	− 5.69/ 8.37	4.23	− 5.99/ 9.59	5.42	−5.26/ 7.05
Kyphosis angle (°) SKW	All groups	46.31	37.89/ 58.35	47.73	39.97/ 58.83	46.92	39.38/ 57.45	45.38	38.72/ 58.11	45.00	40.27/ 58.15	47.00	39.24/ 59.90
	1	42.75	37.79/ 50.69	43.95	40.26/ 50.80	40.18	36.01/ 49.89	40.07	34.25/ 46.14	41.11	36.79/ 49.56	44.17	38.29/ 57.67
	2	45.03	36.76/ 59.72	47.16	38.43/ 58.78	45.86	40.09/ 57.92	44.27	40.44/ 55.11	43.91	41.91/ 51.86	44.16	39.04/ 57.73
	3	55.73	41.99/ 63.41	55.18	46.07/ 66.83	57.17	47.35/ 64.96	58.22	47.03/ 63.04	57.75	42.94/ 63.37	55.00	45.11/ 63.30
Lordosis angle (°) SLW	All groups	24.78	10.20/ 31.03	21.69	10.81/ 36.30	22.04	8.95/ 33.25	20.09	8.11/ 31.21	23.93	12.35/ 32.69	26.06	6.64/ 34.28
	1	19.23	4.29/ 25.83	16.82	9.91/ 31.75	15.95	3.44/ 23.41	14.46	−0.29/ 19.96	14.21	2.46/ 32.89	22.38	2.32/ 39.80
	2	24.78	9.68/ 27.42	18.64	10.38/ 34.39	23.21	9.18/ 35.59	17.69	7.48/ 34.69	23.36	12.53/ 27.91	23.69	4.67/ 33.50
	3	28.02	20.14/ 38.52	32.72	20.73/ 44.32	28.94	15.12/ 39.92	30.46	26.66/ 41.80	27.96	16.61/ 36.92	29.07	8.49/ 34.89

**Table 4:** Working sitting posture. Presentation of median, minimum and maximum of all measurement parameters for all test persons and for each of the three groups isolated. Chair 1: Sirona CARL (Sib), Chair 2: Salli Saddle chair (Sal), Chair 3: Sirona Hugo Freehand (Sio), Chair 4: Aeris Swopper (Sw), Chair 5: KaVo Physioform 5005 (Kg), Chair 6: KaVo Evo (Kb)

Working posture		Chair 1 Sib		Chair 2 Sal		Chair 3 Sio		Chair 4 Sw		Chair 5 kg		Chair 6 Kb	
	Group	Median	Minimum/ Maximum	Median	Minimum/ Maximum	Median	Minimum/ Maximum	Median	Minimum/ Maximum	Median	Minimum/ Maximum	Median	Minimum/ Maximum
**Spine parameter**													
Trunk length D (mm) ARDL	All groups	493.64	472.64/ 521.86	489.99	473.96/ 515.21	491.44	472.31/ 519.09	494.18	471.62/ 515.24	494.10	475.66/ 516.83	496.18	477.57/ 522.61
	1	500.69	479.53/ 531.92	500.34	479.81/ 527.88	493.42	476.57/ 534.37	503.29	472.27/ 533.11	497.45	480.71/ 537.84	499.30	481.10/ 529.98
	2	498.92	474.13/ 525.75	494.09	474.22/ 516.34	500.35	474.57/ 523.64	495.18	473.98/ 526.25	499.26	481.09/ 525.27	505.04	483.02/ 527.36
	3	484.99	466.38/ 497.62	484.53	463.13/ 498.67	481.98	461.52/ 500.07	475.48	460.72/ 498.96	485.25	467.22/ 501.82	484.23	471.88/ 505.21
Trunk length S (mm) ARLS	All groups	534.98	520.59/ 564.10	534.17	517.75/ 556.54	534.16	518.28/ 559.85	534.87	516.31/ 556.82	539.96	522.18/ 564.72	540.70	523.20/ 566.72
	1	534.39	527.93/ 576.00	538.21	523.79/ 569.62	533.36	525.28/ 563.23	534.47	528.41/ 561.17	536.02	526.77/ 571.83	537.43	530.87/ 580.78
	2	550.24	526.57/ 570.71	542.72	522.07/ 567.11	545.09	524.71/ 568.74	547.78	523.18/ 572.96	552.60	529.14/ 570.18	559.31	537.20/ 568.99
	3	523.20	507.16/ 536.38	520.63	507.70/ 537.21	523.02	510.03/ 537.85	522.89	498.68/ 540.82	526.74	514.09/ 541.24	526.02	514.85/ 543.45
Sagittal trunk decline (°) ASRN	All groups	−16.37	−18.33/ −13.69	−15.28	−17.40/ −12.66	−16.25	−18.05/ −13.32	−16.10	−18.87/ −13.23	−16.80	− 19.51/ − 15.17	− 16.67	− 19.72/ − 14.82
	1	− 14.57	− 18.06/ − 12.65	− 15.37	− 18.55/ − 11.82	−14.44	− 17.66/ − 11.57	−14.95	− 17.59/ − 12.06	− 16.02	− 19.20/ − 11.30	− 16.56	− 18.83/ − 13.46
	2	−16.70	− 19.78/ − 14.80	− 14.57	− 16.96/ − 13.00	− 16.32	− 17.95/ − 14.6	−16.04	−20.01/ − 14.45	−16.66	− 18.84/ − 15.28	−17.75	−20.70/ − 15.23
	3	− 17.45	− 19.38/ − 14.36	− 16.63	− 17.40/ − 14.25	−16.33	− 18.94/ − 14.17	−17.54	− 20.65/ − 13.82	−18.26	− 20.59/ − 16.01	− 16.11	− 20.27/ − 14.82
Frontal trunk decline (°) AFRN	All groups	0.99	−0.27/ 1.71	0.66	−0.24/ 1.69	0.46	− 0.15/ 1.64	0.75	− 0.25/ 1.74	1.03	− 0.15/ 1.81	0.66	−0.22/ 1.91
	1	1.25	0.06/ 2.08	0.75	− 0.03/ 1.63	0.69	−0.03/ 1.60	1.09	−0.22/ 1.80	0.66	−0.26/ 2.17	0.73	0.14/ 1.94
	2	1.11	−0.24/ 1.59	0.75	0.16/ 1.84	0.45	−0.05/ 1.64	0.70	−0.19/ 1.64	1.04	0.25/ 1.45	0.03	−0.61/ 1.74
	3	0.70	−1.40/ 1.33	0.51	−0.84/ 2.04	0.21	−0.61/ 2.09	0.36	−0.74/ 1.75	1.29	−1.76/ 2.42	0.99	−0.99/ 1.83
Axis decline (°) AAA	All groups	−0.35	−1.86/ 0.75	−0.75	−1.87/ 0.71	− 0.68	−1.75/ 0.85	−0.56	− 1.94/ 1.06	− 0.27	−1.56/ 0.97	0.01	−1.98/ 0.94
	1	0.28	−1.64/ 1.72	−0.05	−1.62/ 0.88	−0.04	− 1.17/ 1.09	0.34	− 1.55/ 1.52	0.54	− 1.17/ 1.05	0.19	− 1.82/ 1.04
	2	−0.26	−1.58/ 0.69	− 0.77	−2.22/ 0.52	− 0.89	−2.14/ 0.70	−0.93	−1.92/ 0.52	− 0.24	−1.82/ 1.35	−0.19	− 1.84/ 1.08
	3	−0.60	− 2.64/ 0.48	− 0.80	− 2.78/ 0.59	− 1.04	− 2.14/ 0.54	− 0.59	− 2.58/ 0.38	−1.10	− 1.74/ 0.84	−0.21	− 2.34/ 0.73
Thoracic bending angle (°) ATBW	All groups	23.54	19.15/ 27.49	23.44	21.46/ 25.96	22.75	20.12/ 26.56	23.52	20.41/ 27.89	23.62	19.21/ 26.88	23.00	19.69/ 26.85
	1	22.88	19.35/ 27.28	22.80	20.83/ 25.07	21.39	19.78/ 27.24	22.26	19.82/ 23.72	22.59	18.45/ 25.80	21.66	19.35/ 24.22
	2	21.59	17.84/ 25.25	22.83	19.47/ 24.31	22.51	19.18/ 25.02	23.24	20.46/ 25.54	21.67	18.02/ 25.38	20.93	18.78/ 26.05
	3	26.08	21.51/ 30.61	25.92	23.43/ 31.69	25.74	20.88/ 30.63	26.69	21.20/ 31.73	26.88	21.77/ 32.70	26.80	22.63/ 33.24
Lumbar bending angle (°) ALBW	All groups	7.63	4.93/ 10.80	7.40	4.77/ 9.56	8.00	6.21/ 10.50	7.66	5.14/ 10.17	7.19	5.43/ 10.79	7.25	5.54/ 9.28
	1	6.42	4.99/ 10.38	6.49	4.46/ 9.33	7.79	4.76/ 9.21	6.59	3.88/ 9.67	6.97	4.92/ 10.21	6.98	3.83/ 9.07
	2	5.50	4.72/ 10.62	6.84	4.10/ 11.47	6.81	6.26/ 9.30	7.70	5.54/ 11.88	6.56	5.82/ 10.37	5.89	5.21/ 9.22
	3	8.20	6.54/ 10.89	8.23	6.22/ 9.20	9.44	7.36/ 12.86	8.35	6.15/ 12.50	9.65	5.80/ 12.60	8.15	7.10/ 9.68
Standard deviation lateral deviation (mm) ASAS	All groups	3.39	2.32/ 4.10	3.20	2.14/ 4.78	3.20	2.37/ 5.11	3.35	2.58/ 4.71	4.10	2.18/ 5.12	3.51	2.29/ 5.02
	1	3.58	2.00/ 4.00	2.71	2.29/ 4.40	2.87	2.42/ 5.56	3.30	2.25/ 4.65	3.44	1.98/ 5.16	2.72	2.09/ 5.14
	2	3.18	2.32/ 3.98	3.17	2.07/ 5.62	2.83	1.95/ 4.61	3.34	2.70/ 4.39	3.57	2.20/ 4.96	3.43	2.39/ 4.95
	3	3.49	2.76/ 4.62	4.38	2.00/ 5.39	4.02	2.98/ 5.46	4.14	2.37/ 5.27	4.75	2.48/ 5.21	4.19	2.95/ 5.02
Maximal lateral deviation (mm) AMSA	All groups	−3.39	−6.10/ 5.00	−3.87	−6.89/ 3.16	−2.86	−7.14/ 4.52	−1.87	−5.39/ 4.71	−3.90	−7.46/ 6.01	−2.88	−6.41/ 4.86
	1	−3.25	−4.65/ 6.11	−3.83	−5.05/ 4.24	−2.08	−5.23/ 4.95	− 2.46	−4.97/ 4.90	− 2.52	− 6.45/ 6.12	− 3.20	−4.89/ 4.31
	2	−4.15	− 5.82/ 4.22	− 2.64	− 7.75/ 2.67	− 2.32	− 6.71/ 2.77	2.09	− 5.14/ 5.47	− 4.30	− 6.80/ 6.76	0.59	− 6.02/ 5.93
	3	− 5.37	− 6.49/ 5.00	− 5.94	− 7.94/ 3.31	−5.10	−8.07/ 4.85	− 4.72	− 7.07/ 4.10	− 6.62	− 7.84/ 4.33	− 5.47	− 6.83/ 4.73
Standard deviation rotation (°) ASAR	All groups	3.28	2.52/ 4.27	3.48	2.70/ 4.33	3.46	2.26/ 5.00	3.21	2.34/ 4.81	3.26	2.06/ 5.05	3.41	2.56/ 4.50
	1	3.24	2.56/ 3.90	3.21	2.35/ 4.61	3.21	1.98/ 3.89	2.96	1.89/ 3.76	2.20	1.80/ 4.18	3.24	2.06/ 4.30
	2	2.85	2.44/ 4.34	3.21	2.43/ 4.15	3.35	2.59/ 4.88	3.74	2.33/ 4.97	2.94	2.09/ 4.46	3.33	2.15/ 4.34
	3	4.02	2.53/ 4.75	4.10	3.45/ 4.57	4.28	2.26/ 6.07	3.65	2.77/ 4.84	4.31	3.26/ 6.67	4.19	3.16/ 5.42
Maximal rotation (°) AMR	All groups	4.47	−6.74/ 7.52	5.20	−5.82/ 10.14	5.44	−3.99/ 9.06	4.22	−7.40/ 10.32	5.44	−4.14/ 9.10	5.35	− 3.76/ 9.04
	1	5.17	− 7.72/ 6.80	4.16	−5.22/ 9.01	4.70	−3.03/ 7.43	3.16	−6.51/ 8.15	5.64	1.26/ 8.21	6.82	−0.48/ 8.37
	2	5.17	−5.46/ 7.34	3.76	−8.68/ 8.71	6.56	3.90/ 8.70	4.67	−5.20/ 11.32	5.04	−6.83/ 8.95	5.14	−5.25/ 10.07
	3	4.24	−8.09/ 10.77	6.87	−5.29/ 13.42	5.44	−7.24/ 12.46	4.22	−9.33/ 10.23	5.44	−8.22/ 12.13	3.29	−8.81/ 17.16
Kyphosis angle (°) AKW	All groups	57.28	49.61/ 61.59	54.08	48.26/ 64.04	59.06	48.68/ 67.91	55.37	48.10/ 64.10	55.87	46.36/ 68.15	55.72	47.39/ 66.90
	1	52.56	49.52/ 58.24	53.78	46.57/ 56.95	58.40	48.68/ 61.51	52.68	46.78/ 57.01	52.46	49.02/ 57.13	50.27	46.03/ 55.19
	2	49.80	45.77/ 56.59	48.93	42.90/ 59.12	49.97	46.11/ 53.80	49.06	47.37/ 54.89	44.00	40.40/ 51.14	47.44	43.92/ 63.78
	3	65.91	58.48/ 75.27	64.81	54.00/ 77.89	65.77	59.06/ 72.88	65.60	59.92/ 76.42	68.43	59.02/ 74.67	66.90	56.33/ 74.82
Lordosis angle (°) ALW	All groups	27.47	20.16/ 34.5	24.38	21.20/ 36.70	32.27	21.85/ 44.79	28.13	20.98/ 33.8	26.62	16.68/ 41.74	31.33	15.86/ 38.29
	1	24.53	19.39/ 34.03	21.58	18.26/ 30.33	32.06	17.90/ 45.87	24.03	16.30/ 28.24	21.71	16.05/ 32.94	24.49	10.93/ 35.50
	2	24.08	18.92/ 30.44	24.00	21.97/ 31.51	24.47	20.72/ 29.22	26.11	16.79/ 32.48	24.31	5.30/ 40.08	15.32	12.30/ 25.87
	3	32.56	24.63/ 48.62	36.29	21.38/ 53.20	34.92	23.11/ 48.20	33.72	29.69/ 41.28	39.93	23.72/ 46.35	34.99	25.98/ 47.00

### Group comparison of each chair

Table A (supplemental) shows the results from the intergroup comparison per chair, both in the habitual sitting posture and in the working posture. Table 5 provides more detailed information on the significance between the specific groups.

**Table 5:** Group comparison in habitual posture and dental working posture for each chair. Detailed presentation of the Bonferroni-Holm corrected *p-*values of the pair comparison (Wilcoxon-Matched-Pairs test) after significant *p*-values of the Kruskal-Wallis test. Group 1: No dental education, group 2: students of dentistry / dentists < 10 years professional experience, group 3: dentists ≥10 years professional experience

Group comparison	Habitual sitting posture												Working posture											
Chair	1 Sib	*P-*value	2 Sal	*P-*value	3 Sio	*P-*value	4 Sw	*P-*value	5 Kg	*P-*value	6 Kb	*P-*value	1 Sib	*P-*value	2 Sal	*P-*value	3 Sio	*P-*value	4 Sw	*P-*value	5 Kg	*P-*value	6 Kb	*P-*value
**Spine parameter**																								
Trunk length D (mm) RLD	–	–	–	–	–	–	–	–	–	–	–	–	1vs3	0.05	–	–	–	–	–	–	–	–	–	–
Trunk length S (mm) RLS	1vs3 2vs3	0.03 0.02	1vs3 2vs3	0.03 0.03	1vs3 2vs3	0.03 0.03	1vs3 2vs3	0.03 0.02	1vs3 2vs3	0.04 0.03	1vs3 2vs3	0.03 0.03	1vs3 2vs3	0.02 0.01	1vs3 2vs3	0.03 0.03	1vs3 2vs3	0.04 0.02	1vs3 2vs3	0.03 0.01	1vs3 2vs3	0.05 0.03	2vs3	0.01
Frontal trunk decline (°) FRN	1vs3	0.01	–	–	–	–	–	–	–	–	–	–	–	–	–	–	–	–	–	–	–	–	–	–
Thoracic bending angle (°) TBW	–	–	–	–	–	–	–	–	1vs3 2vs3	0.03 0.05	1vs3 2vs3	0.03 0.03	2vs3	0.05	2vs3	0.03	–	–	1vs3	0.04	–	–	1vs3 2vs3	0.04 0.04
Lumbar bending angle (°) LBW	–	–	1vs3 2vs3	0.002 0.02	1vs3	0.02	1vs3 2vs3	0.02 0.03	–	–	–	–	–	–	–	–	–	–	–	–	–	–	–	–
Standard deviation rotation (°) SAR	–	–	–	–	–	–	–	–	–	–	–	–	–	–	–	–	–	–	–	–	1vs3 2vs3	0.001 0.01	–	–
Kyphosis angle (°) KW	–	–	–	–	1vs3	0.001	1vs2 1vs3 2vs3	0.03 0.001 0.05	1vs3	0.03	–	–	1vs3 2vs3	0.01 0.01	1vs3 2vs3	0.01 0.01	–	–	1vs3 2vs3	0.01 0.01	1vs3 2vs3	0.03 0.01	1vs3	0.03
Lordosis angle (°) LW	–	–	1vs3	0.05	1vs3	0.03	1vs3 2vs3	0.001 0.04	–	–	–	–	–	–	–	–	–	–	1vs3	0.02	–	–	–	–
**Shoulder parameter**																								
Scapular height (°) SBS	–	–	–	–	–	–	–	–	–	–	–	–	–	–	–	–	–	–	1vs2	0.02	–	–	–	–
Scapular angle left (°) SWL	–	–	–	–	–	–	–	–	–	–	–	–	–	–	–	–	1vs3 2vs3	0.02 0.02	–	–	–	–	–	–
Scapular angle right (°) SWR	–	–	–	–	–	–	–	–	–	–	–	–	–	–	–	–	1vs3	0.02	–	–	–	–	–	–
**Pelvis parameter**																								
Pelvis torsion (°) BT	–	–	–	–	–	–	–	–	–	–	1vs3 2vs3	0.05 0.05	–	–	–	–	–	–	–	–	–	–	–	–
Pelvis rotation (°) BR	–	–	–	–	–	–	–	–	–	–	–	–	–	–	–	–	–	–	–	–	2vs3	0.03	–	–

#### Habitual sitting posture

The parameter trunk length S was significant for all six chairs (*p* ≤ 0.02). The thoracic bending angle was significant (*p* ≤ 0.02–0.04) for chairs 1, 5 and 6, while the lumbar bending angle was significant in chairs 2, 3, 4 and 5 (*p* ≤ 0.001–0.03). The kyphosis angle was significant for chairs 3, 4 and 5 (*p* ≤ 0.001–0.04), whereas the lordosis angle was significant in chairs 2, 3 and 4 (*p* ≤ 0.001–0.05). The frontal trunk decline was only significant in chair 1 and the maximum lateral deviation was significant only for chair 3 (*p* ≤ 0.01–0.05).

#### Dental working position

As in the habitual sitting posture, the parameter of trunk length S was also significant for all chairs (*p* ≤ 0.01–0.02) in the dental working posture. The parameters of the thoracic bending angle and kyphosis angle showed significances in all chairs, except in chair 3 (*p* ≤ 0.01–0.05). Furthermore, for each parameter only one significance was found: trunk length D in chair 1, sagittal trunk decline in chair 4, standard deviation rotation in chair 5, lordosis angle in chair 4, scapular height in chair 4, scapular angle left and right in chair 3 and pelvis rotation in chair 5 (*p* ≤ 0.001–0.05).

### Inter-chair comparison

All chairs were compared both within the habitual sitting posture and within the working posture. Hence, firstly all groups were tested and, subsequently, each individual group. These data are shown in Table B (supplemental material). Table 6 shows only the significant *p*-values of the multiple pair comparisons following the Friedman tests from Table B (supplemental material).

**Table 6:** Chair comparison. Detailed presentation of the Bonferroni-Holm corrected *p-*values of the pair comparison (Conover-Iman test) after significant Chi^2^-values of the Friedman test. The chair-pairs between which there is a significant difference are shown, together with their *p*-values, first for all subjects and then for each individual group. Chair 1: Sirona CARL (Sib), Chair 2: Salli Saddle chair (Sal), Chair 3: Sirona Hugo Freehand (Sio), Chair 4: Aeris Swopper (Sw), Chair 5: KaVo Physioform 5005 (Kg), Chair 6: KaVo Evo (Kb)

	Habitual sitting posture								Working posture							
Parameter	All groups	P-Wert	Group 1	P-Wert	Group 2	P-Wert	Group 3	P-Wert	All groups	P-Wert	Group 1	P-Wert	Group 2	P-Wert	Group 3	P-Wert
**Spine parameter**																
Trunk length D (mm) RLD	**5 vs. 2** **5 vs. 4** **2 vs. 3** **2 vs. 1** **2 vs. 6** **3 vs. 6** **4 vs. 1** **4 vs. 6**	0.001 0.01 0.01 0.001 0.001 0.01 0.02 0.001	**5 vs. 2** **2 vs. 6**	0.04 0.001	**5 vs. 2** **2 vs. 3** **2 vs. 1** **2 vs. 6** **3 vs. 6** **4 vs. 6**	0.001 0.05 0.001 0.001 0.001 0.001	**–**	–	**5 vs. 2** **5 vs. 3** **5 vs. 4** **2 vs. 6** **3 vs. 6** **4 vs. 6** **1 vs. 6**	0.001 0.03 0.001 0.001 0.001 0.001 0.02	**–**	–	5 vs. 2 2 vs. 1 2 vs. 6 3 vs. 6 4 vs. 6	0.001 0.05 0.001 0.01 0.001	**4 vs. 6**	0.001
Trunk length S (mm) RLS	**5 vs. 2** **5 vs. 4** **2 vs. 3** **2 vs. 1** **2 vs. 6** **3 vs. 6** **4 vs. 1** **4 vs. 6**	0.001 0.01 0.001 0.001 0.001 0.01 0.02 0.001	**5 vs. 2** **2 vs. 6** **4 vs. 6**	0.01 0.001 0.05	**5 vs. 2** **2 vs. 3** **2 vs. 1** **2 vs. 6** **3 vs. 6** **4 vs. 6**	0.001 0.001 0.001 0.001 0.05 0.001	**5 vs. 2** **5 vs. 4**	0.02 0.02	**5 vs. 2** **5 vs. 4** **2 vs. 1** **2 vs. 6** **3 vs. 6** **4 vs. 6** **1 vs. 6**	0.001 0.001 0.03 0.001 0.001 0.001 0.001	**–**	–	**5 vs. 2** **2 vs. 1** **2 vs. 6** **3 vs. 6** **4 vs. 6**	0.001 0.03 0.001 0.02 0.001	**5 vs. 2** **5 vs. 4** **2 vs. 6** **3 vs. 6** **4 vs. 6**	0.03 0.02 0.001 0.03 0.001
Sagittal trunk decline (°) SRN	**5 vs. 2** **2 vs. 3** **2 vs. 4** **2 vs. 1** **2 vs. 6**	0.001 0.001 0.001 0.001 0.001	**2 vs. 3** **3 vs. 4** **3 vs. 1**	0.04 0.05 0.05	**5 vs. 2** **2 vs. 3** **2 vs. 4** **2 vs. 1** **2 vs. 6**	0.001 0.001 0.001 0.001 0.001	**5 vs. 2** **2 vs. 3** **2 vs. 1** **2 vs. 6**	0.001 0.001 0.001 0.02	**5 vs. 2** **5 vs. 3**	0.04 0.04	**–**	–	**2 vs. 6**	0.04	–	–
Axis decline (°) AA	3 vs. 6	0.02	**3 vs. 1**	0.01	–	–	–	–	**5 vs. 3** **3 vs. 4** **3 vs. 1** **3 vs. 6**	0.001 0.02 0.02 0.001	–	–	–	–	–	–
Lumbar bending angle (°) LBW	5 vs. 2 2 vs. 3	0.001 0.02	–	–	–	–	**5 vs. 2** **2 vs. 3** **2 vs. 1**	0.03 0.04 0.04	–	–	–	–	–	–	–	–
Standard deviation rotation (°) SAR	–	–	–	–	–	–	–	–	–	–	–	–	–	–	**5 vs. 2** **5 vs. 3** **5 vs. 4** **5 vs. 1**	0.02 0.05 0.001 0.001
Kyphosis angle (°) KW	**–**	–	–	–	–	–	**5 vs. 2**	0.03	–	–	–	–	–	–	–	–
**Shoulder parameter**																
Scapular distance (mm) SBA	5 vs. 2 2 vs. 3 2 vs. 4 2 vs. 1 2 vs. 6	0.02 0.001 0.01 0.02 0.04	–	–	–	–	–	–	**–**	–	–	–	–	–	–	–
Scapular angle left (°) SWL	**5 vs. 2**	0.05	–	–	–	–	–	–	**2 vs. 3**	0.02	–	–	–	–	–	–
**Pelvis parameter**																
Pelvis distance (mm) BA	2 vs. 1 2 vs. 6	0.04 0.05	–	–	–	–	–	–	**5 vs. 2** **5 vs. 3** **5 vs. 4** **2 vs. 1** **2 vs. 6** **3 vs. 1** **3 vs. 6** **4 vs. 1** **4 vs. 6**	0.04 0.03 0.001 0.04 0.001 0.03 0.001 0.001 0.001	**4 vs. 6**	0.05	**5 vs. 4** **2 vs. 6** **3 vs. 6** **4 vs. 6**	0.02 0.02 0.03 0.001	–	–
Pelvis height (°) BS1	**3 vs. 6** **4 vs. 6**	0.001 0.01	–	–	–	–	–	–	**2 vs. 6** **3 vs. 6**	0.01 0.02	–	–	**5 vs. 6** **2 vs. 6** **4 vs.6**	0.05 0.001 0.01	–	–
Pelvis height (mm) BS2	**3 vs. 6** **4 vs. 6**	0.001 0.01	–	–	–	–	–	–	**2 vs. 6** **3 vs. 6**	0.01 0.03	–	–	**5 vs. 6** **2 vs. 6** **4 vs. 6**	0.05 0.001 0.01	–	–

#### Habitual sitting position

The comparison of the chairs in *all groups* showed significant values for the following parameters: trunk length D, trunk length S, sagittal trunk decline, axis decline, lumbar bending angle, kyphosis angle, scapular distance, scapular angle left, pelvis distance, pelvis height in degrees and pelvis height in mm (*p* ≤ 0.001–0.05). In *group 1*, the trunk length D, trunk length S, sagittal trunk decline and axis decline (*p* ≤ 0.001–0.02) were significant. Significances were found in *group 2* for the trunk length D, trunk length S and sagittal trunk decline (*p* ≤ 0.001). Within *group 3*, differences were found in the trunk length D, trunk length S, sagittal trunk decline, lumbar bending angle, kyphosis angle, pelvis height in degrees and pelvis height in mm (*p* ≤ 0.001–0.05).

#### Dental working position

The following significant differences between the chairs could be observed in *all groups*: trunk length D, trunk length S, sagittal trunk decline, axis decline, scapular distance, scapular angle left, pelvis distance, pelvis height in degrees and pelvis height in mm (*p* ≤ 0.001–0.05). In *Group 1*, only the pelvis distance was significant (*p* ≤ 0.04), while *group 2* had significances for the parameters of trunk length D, trunk length S and sagittal trunk decline, as well as for pelvis distance, pelvis height in degrees and pelvis height in mm (*p* ≤ 0.001–0.05). *Group 3* showed significant deviations in trunk length D, trunk length S, standard deviation rotation and pelvis distance (*p* ≤ 0.03).

### Posture comparison

Table 7 shows the comparison between the *habitual sitting posture* and the *working posture* within each chair. This was calculated, on the one hand, for *all test persons* and, on the other hand, for *each group*. All significances that occurred had a value of *p* ≤ 0.001.

**Table 7:** Posture comparison (habitual vs. working posture) for all parameters related to each chair. Performed once for all subjects and then separately for each group. Chair 1: Sirona (Sib), Chair 2: Salli Saddle chair (Sal), Chair 3: Sirona Hugo Freehand Sio, Chair 4: Aeris Swopper (Sw), Chair 5: KaVo Physioform 5005 (Kg), Chair 6: KaVo Evo (Kb)

Parameter	All Groups						Group 1						Group 2						Group 3					
	1 Sib	2 Sal	3 Sio	4 Sw	5 kg	6 kb	1 Sib	2 Sal	3 Sio	4 Sw	5 Kg	6 Kb	1 Sib	2 Sal	3 Sio	4 Sw	5 kg	6 Kb	1 Sib	2 Sal	3 Sio	4 Sw	5 Kg	6 kb
**Spine parameter**																								
RLD	**0.001**	**0.001**	**0.001**	**0.001**	**0.001**	**0.001**	0.02	**0.001**	**0.001**	**0.001**	0.03	0.09	**0.001**	**0.001**	**0.001**	0.04	**0.001**	**0.001**	0.17	0.02	0.12	0.11	0.04	0.02
RLS	**0.001**	**0.001**	**0.001**	**0.001**	**0.001**	**0.001**	0.01	**0.001**	0.01	0.01	0.01	0.13	**0.001**	**0.001**	**0.001**	0.04	**0.001**	**0.001**	0.03	**0.001**	0.04	0.04	0.01	0.01
SRN	**0.001**	**0.001**	**0.001**	**0.001**	**0.001**	**0.001**	**0.001**	**0.001**	**0.001**	**0.001**	**0.001**	**0.001**	**0.001**	**0.001**	**0.001**	**0.001**	**0.001**	**0.001**	**0.001**	**0.001**	**0.001**	**0.001**	**0.001**	**0.001**
FRN	0.81	0.88	0.47	0.41	0.70	0.99	0.37	0.55	0.62	0.74	0.99	0.90	0.65	0.71	0.28	0.13	0.83	0.23	0.35	0.83	0.80	0.74	0.71	0.13
AA	0.06	0.05	0.17	0.32	0.38	**0.02**	0.37	0.13	0.93	0.35	0.93	0.07	0.70	0.67	0.11	0.09	0.84	0.26	0.11	0.18	0.29	0.33	0.06	0.42
TBW	**0.001**	**0.001**	**0.001**	**0.001**	**0.001**	**0.001**	**0.001**	**0.001**	**0.001**	**0.001**	**0.001**	**0.001**	**0.001**	**0.001**	**0.001**	**0.001**	**0.001**	**0.001**	**0.001**	**0.001**	**0.001**	**0.001**	**0.001**	**0.001**
LBW	0.35	0.05	0.26	0.74	0.39	0.19	0.52	0.45	0.19	0.99	0.35	0.19	0.99	0.93	0.41	0.28	0.29	0.81	0.47	0.02	0.90	0.13	0.95	0.52
SAS	0.29	0.16	0.39	0.74	0.66	0.49	0.20	0.37	0.45	0.25	0.57	0.11	0.57	0.47	0.07	0.74	0.84	0.76	0.89	0.47	0.24	0.89	0.31	0.92
MSA	0.73	0.44	0.43	0.82	0.60	0.63	0.20	0.23	0.23	0.29	0.22	0.67	0.96	0.20	0.35	0.95	0.45	0.99	0.54	0.06	0.10	0.07	0.10	0.10
SAR	0.17	**0.001**	0.01	**0.001**	**0.001**	**0.001**	0.65	0.26	0.33	0.70	0.92	0.35	0.15	0.02	0.20	0.04	0.37	**0.001**	0.49	0.06	0.02	0.02	**0.001**	**0.001**
MR	0.55	0.61	0.12	0.49	0.29	0.40	0.76	0.99	0.35	0.62	0.41	0.45	0.96	0.55	0.14	0.80	0.78	0.93	0.44	0.18	0.80	0.65	0.21	0.49
KW	0.01	**0.001**	**0.001**	**0.001**	**0.001**	0.01	0.02	0.02	**0.001**	**0.001**	**0.001**	0.64	0.88	0.03	0.81	0.31	1.00	0.25	0.08	0.08	0.08	0.10	**0.001**	0.01
**LW**	0.17	**0.001**	0.01	**0.001**	0.10	0.20	0.11	0.04	**0.001**	0.05	0.13	0.84	0.25	0.05	0.63	0.19	0.38	0.50	0.38	0.32	0.21	0.28	0.08	0.38
**Shoulder parameter**																								
SBA	**0.001**	**0.001**	**0.001**	**0.001**	**0.001**	**0.001**	**0.01**	**0.001**	**0.001**	**0.001**	**0.001**	**0.001**	**0.001**	**0.001**	**0.001**	**0.001**	**0.001**	**0.001**	**0.001**	**0.001**	**0.001**	**0.001**	**0.001**	**0.001**
SBS	**0.001**	0.05	0.04	0.01	0.01	0.01	0.01	0.37	0.99	0.60	0.23	0.19	0.01	0.33	0.02	0.01	0.22	0.08	0.37	0.14	0.17	0.16	0.13	0.21
SBR	0.59	0.69	0.74	0.10	0.77	0.76	0.57	0.55	0.43	0.33	0.78	0.52	0.87	0.87	0.78	0.20	0.37	0.76	0.89	0.77	0.83	0.65	0.18	0.95
SWL	0.01	0.01	**0.001**	**0.001**	0.02	0.01	0.18	0.16	0.13	0.06	0.25	0.10	0.04	0.14	0.07	0.16	0.06	0.14	0.24	0.20	**0.001**	0.05	0.35	0.20
SWR	**0.001**	**0.001**	**0.001**	**0.001**	**0.001**	**0.001**	0.04	0.01	0.05	0.02	0.01	0.04	**0.001**	0.05	**0.001**	**0.001**	**0.001**	**0.001**	0.02	0.02	0.03	0.11	0.71	0.47
**Pelvis parameter**																								
BA	0.06	**0.001**	0.05	0.94	**0.001**	0.03	0.37	0.43	0.07	0.55	0.41	0.22	0.29	0.22	0.37	0.49	0.01	0.12	0.18	**0.001**	0.59	0.80	0.07	0.26
BS1	0.50	0.02	0.58	0.62	0.50	0.61	0.96	0.08	0.99	0.31	0.28	0.33	0.70	0.25	0.96	0.68	0.73	0.28	0.09	0.28	0.33	0.95	0.83	0.47
BS2	0.50	0.01	0.57	0.66	0.64	0.56	0.87	0.09	0.96	0.31	0.41	0.31	0.70	0.20	1.00	0.78	0.70	0.28	0.09	0.28	0.42	0.86	0.80	0.44
BT	0.03	0.08	0.15	0.27	0.75	0.81	0.22	0.50	0.62	0.67	0.70	0.25	0.08	0.73	0.18	0.04	0.84	0.60	0.08	0.08	0.26	0.89	0.80	0.02
BR	0.59	0.61	0.41	0.56	0.29	0.22	0.06	0.08	0.86	0.96	0.81	0.60	0.60	1.00	0.76	0.05	0.23	0.31	0.03	0.47	0.23	0.44	**0.001**	0.02

The *posture comparison in all groups* showed statistically significant *p*-values between the habitual sitting and working postures on all six chairs for trunk length D, trunk length S, sagittal trunk decline, thoracic bending angle, scapular distance and scapular angle right.

For chairs 2, 4, 5 and 6, the parameter of standard deviation rotation was significant. The kyphosis angle showed significances for chairs 2, 3, 4 and 5, while the lordosis angle was different in chairs 2 and 4. For the parameter of scapular height, only chair 1 showed a significant difference. In the scapular angle left, significances were found for chairs 3 and 4. Finally, for the parameter of pelvis distance, significance was found for chairs 2 and 5.

Within *group 1* there were deviations in the parameters of sagittal trunk decline, thoracic bending angle and scapular distance for all 6 chairs. Only chairs 2, 3 and 4 differed in the parameter of trunk length D. The parameter of kyphosis angle was significant for chairs 3, 4 and 5, while trunk length S was significant for chair 2 and the lordosis angle for chair 3.

In *group 2*, all six chairs showed significant values for the parameters of sagittal trunk decline, thoracic bending angle and scapular distance. The parameters of trunk length D and trunk length S were significant for all chairs except for chair 4, whereas only in chair 2 did the scapular angle left show no significance. Finally, standard deviation rotation had a significance only in chair 6.

In *group 3*, the parameters of sagittal trunk decline, thoracic bending angle and scapular distance showed significant differences between the habitual sitting and working postures within all chairs used. Standard deviation rotation had significance in chairs 5 and 6. Some parameters had only one significance each: trunk length S for chair 2, kyphosis angle for chair 5, scapular angle left for chair 3, pelvis distance for chair 2 and pelvis rotation for chair 5.

### Subjective assassement of chair comfort

With regard to the group differences of the subjective assessment per chair, no significant group differences are shown in the Chi^2^ test. The corresponding distribution of scores is shown in the form of a contingency table in Table 8.

**Table 8:** Presentation of the subjective evaluation of all chairs according to the school grading system (1 = best). A significant Chi^2^ value is marked with *

	Sib			Sal			Sio			Sw			Kg			Kb		
Notes	G 1	G 2	G 3	G 1	G 2	G 3	G 1	G 2	G 3	G 1	G 2	G 3	G 1	G 2	G 3	G 1	G 2	G 3
1	0	1	1	4	8	6	3	2	0	8	1	3	0	1	2	2	2	2
2	5	6	3	7	7	4	1	2	2	4	11	7	2	5	5	3	6	4
3	5	9	3	2	4	4	9	8	7	2	2	4	13	8	7	11	10	7
4	6	3	5	4	0	1	2	3	4	4	3	0	4	3	2	2	1	5
5	4	0	6	1	1	3	2	4	4	1	1	1	1	0	2	2	1	1
6	0	1	1	2	0	1	3	1	2	1	2	4	0	3	1	0	0	0

## Discussion

The analysis showed that the ergonomic chair layout did not have a clinically relevant effect on the upper body posture, either in the habitual sitting or working postures. There was also no clinically relevant correlation with regard to the choice of profession or the work experience of the dentists. This is confirmed by the subjective assessment of each chair in the group comparison. The sitting position alone, habitual or the anteriorly inclined, dentally-idealized treatment position, was found to be the decisive factor.

Only position-related changes in the sagittal, not in the transverse, plane were observed. The intergroup comparison was carried out separately on each chair in both the habitual sitting and working postures. In general, changes in the sagittal curvature parameters (thoracic bending angle, lumbar bending angle, kyphosis angle and lordosis angle) were found on all chairs and in both sitting positions and were most pronounced in the experienced group of dentists. No rotatory changes took place. The changes in the shoulder and pelvic parameters were not clinically relevant with regard to statistical significance. Therefore, both hypotheses 1 and hypothesis 2 have to be falsified. Despite equal gender distribution in the groups and also similar mean body heights, including standard deviations, the relevant difference in trunk length on all chairs between the groups was due to a more pronounced kyphotic thoracic spine in group 3 (experienced dentists). This could be attributed, independently of the profession, to acquired poor posture due to the older age of the subjects in group 3 [54–56].

In the inter-chair comparison, a harmonious (symmetrical) sitting posture was assumed independently of the chair, both in the habitual and working postures. Only differences in trunk length and sagittal trunk decline were significant in both sitting positions. However, these significances could be attributed to a different ventral inclination of the subjects on the individual chairs, despite the positioning of the subjects with a goniometer at the beginning of measurement taking. In this context, it was found that using the chair pairs 1 and 3, 2 and 4 as well as 5 and 6 result in the most similar posture reactions during sitting. Chairs 1 and 3 were from the same manufacturer, as were chairs 5 and 6. Although chair 2 and chair 4 were based on different ergonomic sitting designs, a similar upper body statics was adopted when sitting.

Overall, the differences of the inter-chair comparison could be classified as minor, since most of the statistical differences were in the range of ±1° or ± 1 mm of measurement error and, thus, a clinical relevance was negligible. Consequently, no change in lordosis and kyphosis angles could be detected when comparing the saddle chair to the other examined chairs. Therefore, hypothesis 3 has to be falsified. The results of Dable et al. [34], Gandavadi et al. [33] and De Bruyne et al. [32] could not be confirmed in this study.

Fiedler [38], who used electromyography to examine muscle activity on similar chairs (Siemens Sirona S, Kavo Physioform 5005, Bambach saddle chair), obtained similar results. He concluded that the chair within a posture has no effect on muscle activity. In the posture comparison (5 different postures that occur during dental work), he was able to demonstrate demonstrable differences in the arm muscles, but hardly any differences for the pelvic and neck muscles. Ellegast et al. [57] also found no differences between four different office chairs and one control chair in terms of posture, muscle activity (M. erector spinae and M. Trapezius) and physical activity intensity in 22 subjects. The different designs of the chairs in their study also had only marginal influences on the subjects, whereas the activity performed (7 office tasks) during the study caused a significant difference in their results. Other studies using EMG [32] and RULA [33, 34] showed that changes in sitting posture did indeed take place on different treatment chairs, although the methods used were different from the videorasterstereography used in this study.

In these studies, the group of saddle chairs was found to cause a straightening of the pelvis and the associated support of the natural lumbar lordosis in comparison to conventional chairs [32, 58, 59], whereas the standard chairs, used as comparative models, showed a reduction in lumbar lordosis and an increased load on the spine and support muscles [32]. The inhomogeneity of the results could also be due to the different measurement methods used; these are difficult to compare with each other and further studies combining the different measurement methods are needed to gain further insight.

In the inter-posture comparison (habitual sitting vs. simulated dental working posture) the sagittal trunk decline, the thoracic bending angle and the shoulder blade distance were comparably significant in all three groups on all chairs, thus, there were no chair-specific differences. It can be confirmed that the habitual sitting posture and the working posture differ significantly in the neck and thoracal area, therefore hypothesis 4 can be verified. However, whether the working posture supports the maintenance of the natural spinal curvature cannot be assessed due to the inhomogeneity of the results of other sagittal curvature parameters. The ventral tilt of the upper body could lead to increased activity of the muscles in the lumbar region and thus favour the development of MSDs. In summary, the habitual sitting posture (averaged over all chairs) (Table 3) can be summarized as a slightly ventrally inclined upper body posture (− 6.95°) with a marginal deviation from the central axis (0.3°). In all groups, the left scapula was positioned about 3.5° further cranially than the right scapula and rotated about 2° posteriorly. The pelvis was almost balanced in all three groups without rotations. The group of experienced dentists had a more pronounced thoracic and lumbar angle. The changes in the simulated working posture could be explained by the instructions in which the test person had to bend ventrally with the upper body and hold an object in front of the sternum [3, 27, 52, 53]; the arms were consequently moved forward resulting in an increased distance between the shoulder blades [26, 60]. All other parameters of the posture comparison were not clinically relevant due to the differences being too small in the data and were, therefore, negligible.

However, the comparison of the two postures using videorasterstereography had some limitations due to the working posture. The ventral inclination of the upper body and the slightly ventrally inclined head meant that the marker on the vertebra prominens could not be recognized in many cases, hence post-marking was necessary. In addition, the lordosis and kyphosis angles were often calculated inadequately due to the anteinclination of the upper body which is why the results, in this regard, should be viewed critically.

Furthermore, the working position adopted during the measurements was only simulated and was not examined in the actual daily work routine; the recording of the upper body statics took only a very short time and had to be carried out under laboratory conditions.

Since this was a cross-sectional analysis carried out under laboratory conditions, the long-term influence of the chairs used on the sitting posture should be investigated in future studies and under real-life conditions. Studies with prolonged data cohorts in an occupational environment could establish the chair’s impacts on the occupational environment’s exposure activities in future. Despite the different ergonomic sitting layouts of the dentist’s chairs used, no significant measurable improvement in dental workplace ergonomics could be achieved. Although no measurable positive changes in upper body statics could be demonstrated, this does not mean that individual MSDs could not be reduced or prevented by one of these chairs, as discomfort is related to subjective perception. As in other studies in which different postures showed significant changes in muscle activity, joint angles, and physical activity, the results of this study have led to the conclusion that working posture has a crucial influence on the upper body posture of both dentists [15, 21, 38] and non-dentists [57]. Since differences between the habitual and the dentist-oriented sitting posture were found in this study, the working posture in particular should be considered as a starting point for further modifications of the chairs. Subsequently, working posture should also become more important for improving workplace ergonomics in dentistry. Attention should be paid to an upright, neutral and balanced working posture in order to minimize stress and thus counteract overload and its consequences in the long term [13, 61]. As a recommendation, working in a seated position should not be chosen as the sole and permanent working position, but a varied and dynamic way of working, avoiding long lasting static positions, should be integrated into the daily routine in order to minimize the risk of developing work-related MSDs [29, 62, 63].

## Conclusions

The results showed that neither the ergonomic chair layout had any clinically relevant effect on the upper body posture, nor could a clear subjective improvement be confirmed by a particular chair. The working posture preferably adopted by the dentist may have a greater impact on the development of some musculoskeletal diseases than the chair ergonomics. The selection of the dentist’s chair contributes to a small extent (if at all) to the improvement of ergonomics in the daily dental work routine. Consequently, work related MSDs are traced back to multi-causal reasons [13, 18, 23, 30]. Therefore, the dentists’s chair selection via its ergonomic aspects should be regarded as a constituent part in the overall workplace ergonomics.

## Supplementary Information

**Additional file 1: Table A**. Group comparison. *P*-values of the Kruskal-Wallis test for each chair; firstly, for the habitual sitting posture and then for the dental working posture. **Table B**. Chair comparison. Chi^2^-values of the Friedman test; first for all test persons together and then isolated for each group.

## Data Availability

All data generated or analysed during this study are included in this published article and its supplementary information files.
