# Supplementary material for: Influence of design of dentist’s chairs on body posture for dentists with different working experience
Source: BMC Musculoskelet Disord. 2021 May 19;22:462. doi: 10.1186/s12891-021-04334-1 (PMC8136039; doi:10.1186/s12891-021-04334-1)
Supplement: Supplementary file 1 — Additional file 1: Table A. Group comparison. P-values of the Kruskal-Wallis test for each chair; firstly, for the habitual sitting posture and then for the dental working posture. Table B. Chair comparison. Chi2-values of the Friedman test; first for all test persons together and then isolated for each group. [file 12891_2021_4334_MOESM1_ESM.docx]

**Influence of design of dentist’s** **chairs on body posture for dentists with different working experience**

**Huppert F**^1^**, Betz W**^2^**, Maurer-Grubinger C**^1^**, Holzgreve F**^1*^**, Fraeulin L**^1^**, Filmann N³, Groneberg DA**^1^**, Ohlendorf D**^1^

^1^ Institute of Occupational Medicine, Social Medicine and Environmental Medicine, Goethe-University Frankfurt/Main, Theodor-Stern-Kai 7, Building 9A, Frankfurt/Main 60590, Germany

^2^ Institute of Dentistry, Goethe-University, Frankfurt/Main, Theodor-Stern-Kai 7, Frankfurt am Main 60590, Germany

^3^ Institute of Biostatistics and Mathematical Modeling, Goethe-University, Frankfurt/Main, Theodor-Stern-Kai 7, Building 11, Frankfurt/Main 60590, Germany

Supplemental material

| **Group comparison** | **Habitual sitting posture** | | | | | | **Working posture** | | | | | |
| --- | --- | --- | --- | --- | --- | --- | --- | --- | --- | --- | --- | --- |
| **Chair** | 1 Sib | 2 Sal | 3 Sio | 4 Sw | 5 Kg | 6 Kb | 1 Sib | 2 Sal | 3 Sio | 4 Sw | 5 Kg | 6 Kb |
| **Spine parameter** | | | | | | | | | | | | |
| Trunk length D (mm) RLD | 0.10 | 0.13 | 0.08 | 0.08 | 0.15 | 0.20 | **0.05** | 0.09 | 0.07 | 0.07 | 0.12 | 0.10 |
| Trunk length S (mm)  RLS | **0.02** | **0.02** | **0.02** | **0.02** | **0.02** | **0.02** | **0.01** | **0.02** | **0.01** | **0.01** | **0.02** | **0.02** |
| Sagittal trunk decline (°)  SRN | 0.33 | 0.89 | 0.88 | 0.63 | 0.31 | 0.66 | 0.11 | 0.50 | 0.17 | **0.05** | 0.17 | 0.29 |
| Frontal trunk decline (°)  FRN | **0.01** | 0.64 | 0.37 | 0.16 | 0.70 | 0.40 | 0.26 | 0.94 | 0.63 | 0.68 | 0.99 | 0.34 |
| Axis decline (°)  AA | 0.10 | 0.65 | 0.38 | 0.27 | 0.42 | 0.43 | 0.23 | 0.59 | 0.25 | 0.24 | 0.35 | 0.73 |
| Thoracic bending angle (°)  TBW | **0.04** | 0.11 | 0.24 | 0.06 | **0.03** | **0.02** | **0.05** | **0.02** | 0.15 | **0.03** | **0.03** | **0.02** |
| Lumbar bending angle  (°)  LBW | 0.15 | **0.001** | **0.02** | **0.01** | **0.03** | 0.34 | 0.17 | 0.46 | 0.09 | 0.26 | 0.20 | 0.16 |
| Standard deviation lateral deviation (mm)  SAS | 0.95 | 0.85 | 0.67 | 0.81 | 0.99 | 1.00 | 0.51 | 0.50 | 0.21 | 0.73 | 0.54 | 0.35 |
| Maximal lateral deviation (mm)  MSA | 0.86 | 0.14 | **0.05** | 0.08 | 0.07 | 0.32 | 0.68 | 0.55 | 0.63 | 0.37 | 0.81 | 0.66 |
| Standard deviation rotation (°)  SAR | 0.79 | 0.44 | 0.44 | 0.22 | 0.34 | 0.08 | 0.46 | 0.16 | 0.16 | 0.06 | **0.001** | 0.16 |
| Maximal rotation (°)  MR | 0.74 | 0.83 | 0.32 | 0.22 | 0.56 | 0.90 | 0.96 | 0.50 | 0.71 | 0.68 | 0.74 | 0.91 |
| Kyphosis angle (°)  KW | 0.12 | 0.07 | **0.01** | **0.001** | **0.04** | 0.15 | **0.01** | **0.01** | 0.08 | **0.01** | **0.01** | **0.03** |
| Lordosis angle (°)  LW | 0.09 | **0.05** | **0.04** | **0.001** | 0.20 | 0.41 | 0.16 | 0.13 | 0.27 | **0.03** | 0.22 | 0.08 |
| **Shoulder parameter** | | | | | | | | | | | | |
| Scapular distance (mm)  SBA | 0.35 | 0.27 | 0.37 | 0.37 | 0.45 | 0.38 | 0.50 | 0.36 | 0.42 | 0.52 | 0.46 | 0.59 |
| Scapular height (°)  SBS | 0.09 | 0.24 | 0.18 | 0.26 | 0.14 | 0.11 | 0.11 | 0.18 | 0.07 | **0.03** | 0.13 | 0.17 |
| Scapular rotation (°)  SBR | 0.79 | 0.65 | 0.26 | 0.18 | 0.27 | 0.78 | 0.58 | 0.73 | 0.47 | 0.49 | 0.29 | 0.97 |
| Scapular angle left (°)  SWL | 0.27 | 0.18 | 0.34 | 0.11 | 0.21 | 0.21 | 0.66 | 0.26 | **0.01** | 0.10 | 0.14 | 0.58 |
| Scapular angle right (°)  SWR | 0.21 | 0.22 | 0.47 | 0.14 | 0.08 | 0.27 | 0.11 | 0.72 | **0.03** | 0.33 | 0.81 | 0.41 |
| **Pelvis parameter** | | | | | | | | | | | | |
| Pelvis distance (mm)  BA | 0.94 | 0.97 | 0.95 | 0.96 | 0.96 | 0.59 | 0.98 | 0.99 | 0.94 | 0.96 | 1.00 | 0.90 |
| Pelvis height (°)  BS1 | 0.89 | 0.78 | 0.97 | 0.87 | 0.97 | 0.95 | 0.77 | 0.89 | 0.69 | 0.66 | 0.75 | 0.75 |
| Pelvis height (mm)  BS2 | 0.95 | 0.83 | 0.98 | 0.88 | 0.93 | 0.93 | 0.74 | 0.92 | 0.60 | 0.71 | 0.78 | 0.66 |
| Pelvis torsion (°)  BT | 0.07 | 0.09 | 0.08 | 0.14 | 0.49 | **0.03** | 0.21 | 0.52 | 0.74 | 0.10 | 0.52 | 0.86 |
| Pelvis rotation (°)  BR | 0.41 | 0.90 | 0.88 | 0.80 | 0.97 | 0.58 | 0.81 | 0.36 | 0.80 | 0.56 | **0.04** | 0.13 |

Table A: Group comparison. P-values of the Kruskal-Wallis test for each chair; firstly, for the habitual sitting posture and then for the dental working posture.

|  | **Habitual sitting posture** | | | | **Working position** | | | |
| --- | --- | --- | --- | --- | --- | --- | --- | --- |
| **Parameter** | All Groups | Group 1 | Group 2 | Group 3 | All Groups | Group 1 | Group 2 | Group 3 |
| **Spine parameter** | | | | | | | | |
| Trunk length D (mm)  RLD | **0.001** | **0.01** | **0.001** | **0.05** | **0.001** | 0.25 | **0.001** | **0.01** |
| Trunk length S (mm)  RLS | **0.001** | **0.001** | **0.001** | **0.01** | **0.001** | 0.13 | **0.001** | **0.001** |
| Sagittal trunk decline (°)  SRN | **0.001** | **0.001** | **0.001** | **0.001** | **0.001** | 0.13 | **0.05** | 0.38 |
| Frontal trunk decline (°)  FRN | 0.35 | 0.52 | 0.62 | 0.59 | 0.92 | 0.99 | 0.66 | 0.73 |
| Axis decline (°)  AA | **0.02** | **0.02** | 0.72 | 0.25 | **0.001** | 0.24 | 0.13 | 0.08 |
| Thoracic bending angle (°)  TBW | 0.06 | 0.15 | 0.30 | 0.23 | 0.85 | 0.27 | 0.71 | 0.43 |
| Lumbar bending angle (°)  LBW | **0.01** | 0.16 | 0.45 | **0.01** | 0.30 | 0.75 | 0.40 | 0.29 |
| Standard deviation lateral deviation (mm)  SAS | 0.53 | 0.80 | 0.48 | 0.55 | 0.37 | 0.86 | 0.52 | 0.33 |
| Maximal lateral deviation (mm)  MSA | 0.36 | 0.08 | 0.81 | 0.37 | 0.14 | 0.98 | 0.10 | 0.54 |
| Standard deviation rotation (°)  SAR | 0.55 | 0.74 | 0.33 | 0.77 | 0.30 | 0.50 | 0.51 | **0.001** |
| Maximal rotation (°)  MR | 0.14 | 0.64 | 0.58 | 0.21 | 0.27 | 0.36 | 0.38 | 0.22 |
| Kyphosis angle (°)  KW | **0.05** | 0.66 | 0.66 | **0.05** | 0.68 | 0.55 | -- | 0.67 |
| Lordosis angle (°)  LW | 0.17 | 0.32 | 0.80 | 0.20 | 0.28 | 0.55 | -- | 0.36 |
| **Shoulder parameter** | | | | | | | | |
| Scapular distance (mm)  SBA | **0.001** | 0.45 | 0.06 | 0.06 | **0.05** | 0.30 | 0.34 | 0.74 |
| Scapular height (°)  SBS | 0.66 | 0.36 | 0.91 | 1.00 | 0.27 | 0.64 | 0.25 | 0.57 |
| Scapular rotation (°)  SBR | 0.69 | 0.74 | 0.92 | 0.37 | 0.65 | 0.69 | 0.65 | 0.17 |
| Scapular angle left (°)  SWL | **0.05** | 0.38 | 0.64 | 0.20 | **0.04** | 0.40 | 0.17 | 0.06 |
| Scapular angle right (°)  SWR | 0.70 | 0.82 | 0.81 | 0.16 | 0.55 | 0.85 | 0.18 | 0.93 |
| **Pelvis parameter** | | | | | | | | |
| Pelvis distance (mm)  BA | **0.01** | 0.27 | 0.20 | 0.12 | **0.001** | **0.04** | **0.001** | **0.03** |
| Pelvis height (°)  BS1 | **0.001** | 0.54 | 0.37 | **0.05** | **0.001** | 0.90 | **0.001** | 0.11 |
| Pelvis height (mm)  BS2 | **0.001** | 0.64 | 0.38 | **0.05** | **0.01** | 0.77 | **0.001** | 0.12 |
| Pelvis torsion (°)  BT | 0.40 | 0.56 | 0.81 | 0.57 | 0.37 | 0.78 | 0.36 | 0.44 |
| Pelvis rotation (°)  BR | 0.78 | 0.70 | 0.80 | 0.37 | 0.25 | 0.46 | 0.19 | **0.02** |

Table B: Chair comparison. Chi²-values of the Friedman test; first for all test persons together and then isolated for each group.
